# What shapes gender attitudes among adolescent girls and boys? Evidence from the UDAYA Longitudinal Study in India

**DOI:** 10.1371/journal.pone.0248766

**Published:** 2021-03-18

**Authors:** Sangram Kishor Patel, K. G. Santhya, Nicole Haberland

**Affiliations:** 1 Population Council, New Delhi, India; 2 Population Council, New York, NY, United States of America; Institute of Economic Growth, INDIA

## Abstract

**Introduction:**

The role of gender norms in shaping education and work opportunities, distribution of power and resources, and health and wellbeing is well recognised. However, rigorous studies in low- and middle-income countries on when and how norms change over time and what factors shape adolescents’ and young adult’s gender attitudes are limited. This paper explores the factors that determine adolescents’ gender attitudes, as well as patterns in gender attitude shifts over time among younger and older adolescent boys and girls in India.

**Data and methods:**

Data presented in this paper were drawn from a unique longitudinal study of adolescents aged 10–19 (Understanding the lives of adolescent and young adults–UDAYA study) in the states of Bihar and Uttar Pradesh in India, conducted during 2015–2016 (wave 1) and 2018–2019 (wave 2). The analysis presented in this paper drew on data from 4,428 boys and 7,607 girls who were aged 10–19 and unmarried at wave 1 and interviewed at both rounds of the survey. We used univariate and bivariate analyses to examine changes in adolescents’ gender role attitudes over time and the association between explanatory variables and gender role attitudes. We also used linear fixed effects regression models to identify factors that shape adolescents’ gender role attitudes.

**Results:**

Gender role attitudes became more egalitarian over time among boys and girls, except among the older cohort of boys in our study. Among both younger and older cohorts, girls/young women held more egalitarian views than boys/young men and this pattern held over time for both cohorts. Factors that influenced gender role attitudes differed for younger and older adolescents, particularly among boys. While some predictors differed for boys and girls, there were substantial similarities as well. Gender attitudes were affected by factors at the individual, family, peer, and societal levels, as well as by community engagement.

**Conclusions:**

Our findings show that it is possible to shift gender attitudes toward greater equity and, in so doing, contribute to improved health and rights.

## Introduction

Gender norms have long been recognized as shaping education and work opportunities, distribution of power and resources, and health and wellbeing [1–3]. Defined as “often unspoken rules that govern the attributes and behaviors that are valued and considered acceptable for men, women, and gender minorities” [3], gender norms are often measured by assessing individuals’ gender attitudes, or their views on gender norms, as an approximation of norms. And, indeed, inequitable gender attitudes have been linked with adverse maternal and child health outcomes, inadequate child nutrition, lower rates of contraceptive use, and experience and perpetration of intimate partner violence, among other outcomes [4–10].

While gender norms are reinforced and reproduced over the life course by individuals, family, peers, media, schools, religion, and other structural factors, norms can and do change. However, questions about when views on gender norms are more open to change–for example, are they more mutable in early or late adolescence?–remain unanswered, despite their import for program design. Recent longitudinal studies from the U.S. show that, in general, male and female gender attitudes became more equitable over the course of adolescence with variation by factors such as country of birth, birth order, age, sex, and parents’ gender attitudes [11–13]. To our knowledge, only one study has explored this question with longitudinal data from a low income country, finding that at the aggregate level Zambian adolescent girls’ gender attitudes became slightly more equitable over time, but that this shift was significant only among urban girls [14].

Also of import to programs is understanding what forces drive gender attitudes in which direction–for example, are schools a progressive influence or do they reinforce restrictive norms? Theoretical frameworks frequently take an ecological or systems approach that considers factors at multiple levels: individual, relational, social, institutional, and structural. These include, for example, the influence of parents, peers, relationships, school, media, workplace culture, laws and policies [1, 15, 16]. A recent systematic review documents links between gender attitudes and interpersonal and community factors, finding relatively consistent evidence of an association between gender attitudes and family and peer influence, and inconclusive evidence of an association with media and schools [17]. However, the vast majority of studies in the review used cross-sectional study designs, precluding any conclusions about causality. Longitudinal studies that identify the factors that shape young people’s gender attitudes are needed.

In India, inequitable gender norms and attitudes among adolescents and children are increasingly explored in research. Recent studies observe that adherence to unequal gender norms was evident even among young adolescents and that girls consistently reported more equitable attitudes than boys [18–21]. Shukla (2015) documenting inter-generational differences in gender role attitudes notes that attitudes were generally more equitable among younger generations, yet some norms, especially those related to dowry, violence against women, and women’s right to inheritance, were resistant to change [20]. Landry and colleagues (2020) examined differences in gender attitudes among girls and boys in grades 5 through 9 in Delhi, Punjab, and Rajasthan, finding that adolescents in higher grades reported more equitable attitudes than those in lower grades [21]. However, all these studies used cross-sectional data, leaving open the questions of whether, when, and how norms change over time.

Some of the few studies that rigorously assessed the formation of gender attitudes in India have, for example, documented the role of parents, especially mothers, in shaping children’s gender attitudes [22, 23] and the effect of television on shaping more gender equitable attitudes [24]. Randomized trials of interventions that aim to shift gender norms confirm that gender attitudes are mutable. For example, a study in Bihar, India, shows that exposure to gender-transformative life skills education and sports-coaching programme helped boys accept egalitarian gender role attitudes and notions of masculinity, and reject attitudes about men’s controlling behaviours over women/girls and perpetration of violence [25]. Notably, the evaluation found that the program had greater effect sizes among younger boys (13–14) than older boys (15–19), although significant improvements were detected among both younger and older boys [26]. A 12-month non-randomized pre-post evaluation of a programme that sought to instill gender-equitable attitudes among adolescent male cricket athletes by sensitizing their coaches in schools in Mumbai, India found that gender attitudes were more equitable among boys whose coaches participated in the intervention as compared to those whose coaches did not [27]. An evaluation study of a multi-year school-based intervention in Haryana, India, that engaged adolescents in classroom discussions about gender equality shows that program participants reported more gender-equitable behavior; for example, boys reported helping out more with household chores [23].

How gender attitudes are constructed during adolescence in the absence of gender-focused interventions is less well understood. In this paper, we use a unique longitudinal data set from India–Understanding the lives of adolescent and young adults study–hereafter referred to as UDAYA study–to explore the factors that determine adolescents’ gender attitudes, as well as patterns in gender attitude shifts over time among younger and older adolescent boys and girls.

## Data and methods

### Study setting

The study was conducted in the states of Bihar and Uttar Pradesh in India. Uttar Pradesh, with a population of 199.8 million in 2011, has the largest population of any state in the country, accounting for 17 percent of India’s population [28]. Bihar, with a population of 104.1 million, is the third largest state in terms of population, accounting for nine percent of India’s population. Both states are predominantly rural with just 11 percent of the population in Bihar and 22 percent in Uttar Pradesh residing in urban areas in 2011. Economically, Bihar and Uttar Pradesh are the poorest among all the states and union territories in India with per capita income well below the national average [29] and 34 percent of the population in Bihar and 29 percent in Uttar Pradesh were estimated to be living below the poverty line in 2011–12 [30].

Gender inequality and harmful gender norms and practices are prevalent in both states. For example, the sex ratios are skewed against females and are lower than that for India as a whole (918 females in Bihar, 912 females in Uttar Pradesh and 943 females in India per 1,000 males in 2011 [28]. There is a strong preference for sons; 31–37% of women and 28–30% of men expressed a desire to have more sons than daughters, but only 1–4% wanted more daughters than sons in these states in 2015–16 [31, 32]. Females are less literate than males in both states (literacy rate of 71% for males versus 52% for females in Bihar and 77% for males versus 57% for females in Uttar Pradesh in 2011 [28]. Labour force participation is considerably lower among females than males in both states– 20–25% of women compared with 76–79% of men aged 15–49 were employed in the 12 months preceding the survey in 2015–16. Early and child marriage among girls is prevalent in both states, particularly in Bihar– 42% of women aged 20–24 years in Bihar and 21% in Uttar Pradesh were married before age 18 [31, 32]. Large proportions of women and men justify violence against women and girls. In Bihar, for example, 53% of women and 38% of men believed it is justifiable for a husband to beat his wife under selected circumstances as did 51% of women and 42% of men in Uttar Pradesh [31, 32]. Some 34–39% of women aged 15–49 had ever experienced physical or sexual violence.

Bihar and Uttar Pradesh, with 72 million adolescents aged 10–19, are home to every fourth adolescent in India [28]. Available evidence highlights that the situation of adolescents in these states is characterised by poor levels of educational attainment, with wide differences by gender in enrolment, substantial levels of unemployment, considerable vulnerability in sexual and reproductive health matters, limited agency, particularly among adolescent girls, and limited access to health services [33, 34].

### Study design and study population

Data presented in this paper were drawn from a unique longitudinal study of adolescents aged 10–19 (Understanding the lives of adolescent and young adults study–hereafter referred to as UDAYA study) in Bihar and Uttar Pradesh. The first wave was conducted in 2015–2016, and the follow up survey was conducted three years later in 2018–2019. The 2015–16 survey was conducted among a state-representative sample of unmarried boys and girls aged 10–19 and married girls aged 15–19. The study adopted a systematic, multi-stage stratified sampling design to draw sample areas independently for rural and urban areas. A total of 150 primary sampling units (PSUs)—villages in rural areas and census wards in urban areas—were selected in each state, using the 2011 census list of villages and wards as the sampling frame. In each primary sampling unit (PSU), households to be interviewed were selected by systematic sampling. A complete mapping and household listing operation was carried out in each selected PSU (or in selected segments or linked villages as appropriate). Based on this list, a PSU was divided into two nearly equal segments where one segment was randomly chosen for conducting interviews of females and the other for interviews of males (Married girls were interviewed from both male and female segments). The list of households within each segment provided the necessary frame for selecting households within the segment. Within each selected household, no more than one respondent per category was interviewed, which resulted in a maximum of three interviews from any household—one younger girl, one unmarried older girl, and one married older girl in the female segment, and one younger boy, one older boy, and one married older girl in the male segment. In case more than one respondent from a single category was found in the household, one respondent was selected randomly using the Kish table. No replacement of the respondent thus selected was allowed (for more details about the study design, see [33, 34]. Using a structured questionnaire, the field investigators interviewed 20,594 adolescents; response rate for the survey was 92%, and 1% of selected respondents refused to participate. The main reason for non-response was that the respondent was not at home (5%).

**Fig 1:**
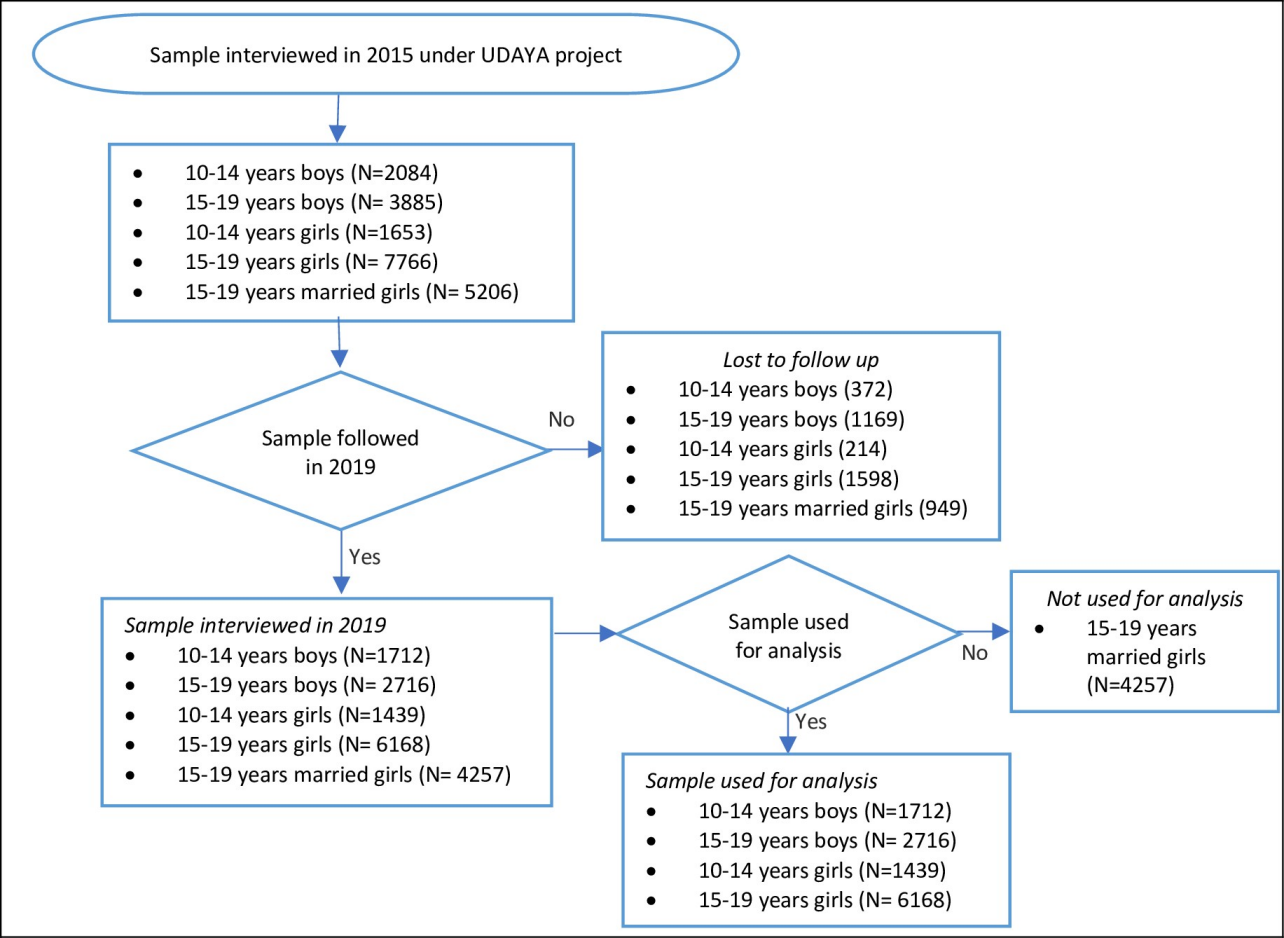
Flowchart on the final sample used for the analysis in this study.

In 2018–19, we interviewed again those who were successfully interviewed in 2015–16, and who consented to be re-interviewed. Of the 20,594 who were eligible for re-interview, we re-interviewed 4,567 boys and 12,251 girls. We excluded respondents (3%) who gave inconsistent response to questions related to age and education at the follow-up survey; therefore, the final follow-up sample comprised 4,428 boys and 11,864 girls, thus resulting in an effective follow-up rate of 74% for boys and 81% for girls. The main reasons for loss-to-follow-up were that the participant had migrated (10% for boys and 6% for girls), and the participant or his/her parent or guardian refused (7% for boys and 6% for girls). We note that the characteristics of those who were re-interviewed and those who could not be re-interviewed differed significantly in terms of age, education, place of residence, caste, and religion (see S1 Table for attrition bias). Of these variables, age and education were controlled in the fixed effects regression model and caste and religion were omitted as these characteristics were time invariant. The analysis presented in this paper drew on data from the subset of 12,035 adolescents who were aged 10–19 and unmarried at wave 1 (4,428 boys and 7,607 girls) (see Fig 1); girls who were aged 15–19 and married at wave 1 were excluded.

### Ethics statement

The study protocol was approved by the Institutional Review Board of the Population Council. We took several measures to ensure that research ethics was strictly followed. Interviews of boys and girls were undertaken in separate segments of each primary sampling unit to avoid any risk of teasing, harassment and harm to girls’ reputation if interviews of boys and girls were conducted in the same geographical segments. Interviews were conducted separately but simultaneously in case more than one respondent was selected from a household. In order to minimise discomfort during questioning, the scenarios and terminologies described by adolescents were adapted for use in our questionnaire on sensitive topics. Based on our earlier experiences of working with young adolescents, we made the survey questions age-appropriate—for example, we did not ask about sexual and reproductive health matters with young adolescents. Interviewers underwent extensive training in ethical issues, and teams were instructed to apprise community leaders about the study and seek their support for its implementation in the community. Consent was sought from each individual to be interviewed, and for unmarried adolescents aged 10–17, consent was also sought from a parent or guardian. Names were never recorded in the computer form in which data were collected. In order to preserve the confidentiality of the respondent or the parent/guardian, signing the consent form was optional; however, the interviewer was required to sign a statement that she or he had explained the content of the consent form to the respondent or parent. Interviewers were instructed to skip to relatively non-sensitive sections in case the interview was observed by parents or other family members, call upon a fellow interviewer to conduct parallel discussion sessions with bystander, conduct interviews in locations that offered privacy for the interview and terminate interviews if privacy could not be ensured. Finally, the study team approached NGOs that conduct youth or health-related activities at the district level, help lines that work at national or sub-national levels and public health authorities and referred study participants in need of information or services.

### Variables

#### Outcome variable

The outcome variable in our analysis was an index of gender role attitudes (GRA). We asked a number of age-appropriate questions to respondents to assess their gender role attitudes at both waves of the survey; however, we used those questions that were posed at both waves to each category of respondents (i.e., younger and older adolescents) in order to ensure the comparability of the index for younger and older adolescents over time. These questions included, for adolescents who were aged 10–14 at wave 1, whether: (1) it is more important to educate boys than girls, (2) boys should do as much domestic work as girls, (3) girls like to be teased by boys, (4) girls should be allowed to decide when they want to marry, and (5) fathers should mainly decide how household money should be spent. For adolescents who were aged 15–19 at wave 1, the questions were whether: (1) it is wrong for a girl to have male friends, (2) girls like to be teased by boys, (3) girls should be allowed to decide when they want to marry, (4) the husband should mainly decide how household money should be spent, and (5) bathing and feeding children were women’s responsibility only. We created an additive index that summarised the participants’ responses to these questions, giving a score of 1 for each gender egalitarian response and 0 otherwise. Those who responded with the option of ‘don’t know/cannot say’ were combined with 0 (0.4–5% of respondents). The index was created separately for those who were aged 10–14 and 15–19 at wave 1; the value of the index ranged from 0 indicating inequitable views to 5 indicating adherence to high egalitarian attitudes (alpha was 0.52 for younger adolescents and 0.54 for older adolescents).

#### Explanatory variables

Our analysis explored the role of factors that have been hypothesized to shape gender attitudes and norms. These included: individual-level factors (age, aspirations); family-level influence (participants’ communication with their parents, experience of gender discriminatory practices at home, and household wealth); peer influence (peer network size, role models); community engagement/participation such as group membership (e.g. self-help groups, sports clubs, etc.), political participation, participation in adolescent programs (such as family life education, adolescent health days, etc.), interaction with frontline workers (Accredited Social Health Activists and Anganwadi workers); and societal institutions and influences including education (educational attainment), work (paid work in past year), and digital media (internet or social media). Table 1 provides a brief description of each variable and Table 2 shows the summary statistics at wave 1 and wave 2 for the explanatory variables used in the analysis.

**Table 1:** Description for explanatory variables used in the analysis.

Variables	Definition and response categories
**Individual-level variables**	
Age	In completed years
Aspirations	Thought about the profession or vocation respondent likes to follow when s/he reaches adulthood (Yes/No).
	Question to capture future aspirations was phrased slightly differently at wave 1 (‘what would you like to become in future?’) and at wave 2 (‘In future, what kind of work do you prefer to engage in for earning money?’). All those who responded with a vocation or profession (such as, teacher/doctor/engineer/scientist/lawyer or join police force/army or start a business or work in informal sectors like tailor, plumber, electrician, carpenter, etc.) were categorized as having thought about future aspiration and those who replied that they won’t work, can’t say or haven’t thought about it were categorized as “no”.
**Family-level variables**	
Parent-child communication	Discussed the following topics (school performance, friendship, experience of bullying, physical changes during adolescence or how pregnancy occurs) with mother or father in the year preceding the interview (Yes/No). Question about reproductive processes, i.e., how pregnancy occurs was asked to those aged 15 and above only. Respondents who discussed any one of the topics with either father or mother were coded as “yes” and respondents who discussed none of the topics with their father or mother were coded as “no”.
Gender discriminatory experience at home	Experienced gender discriminatory practices at home where parents favoured sons over daughters in any of the following situations (the quantity or quality of food items given, the amount of pocket money given, the type of school in which they were enrolled, and parental aspirations for the respondent’s education) (Yes/No). Questions on gender discriminatory practices at home, i.e., whether their parents favoured them (among boys) or discriminated against them (among girls) vis-à-vis their opposite-sex siblings, were posed to those who reported co-residing with opposite-sex siblings who were up to three years younger or older than the respondent; those respondents who were not eligible for these questions were considered not to have experienced gender discriminatory practices. Respondents who reported discrimination in any one of the situations were coded as “yes” and respondents who reported discrimination in none of the situation were coded as “no”.
Household wealth	Household wealth index based on ownership of selected durable goods and amenities with possible scores ranging from 0 to 57; households were then divided into quintiles, with the first quintile representing households of the poorest wealth status and the fifth quintile representing households with the wealthiest status.
**Peer influence related variables**	
Peer network size	Reported five or more friends (Yes/ No).
Role models	Reported having a role model (Yes/No). The role models reported were family members/relatives, teachers, professionals, friends, army/police, sports personalities, friends, actors, politicians and others.
**Community engagement/participation related variables**	
Group membership	Reported membership in any organised groups such as self-help groups, sports clubs, groups formed under government programmes (e.g., Kishori Shakti Yojana, SABLA, Nehru Yuva Kendra Sangathan) or any other groups (Yes/No). Question on group membership was phrased slightly differently at wave 1 (‘are you member of a group formed under Kishori Shakti Yojana, SABLA, Nehru Yuvak Kendra Sangathan, any self-help groups, sports clubs or any other groups?’) and at wave 2 (‘Have you been a member of any adolescent/youth groups such as groups formed under Kishori Shakti Yojana/SABLA, Nehru Yuvak Sangathan, self-help groups, sports groups in the last three years?’)
Political participation	Reported membership in a political party or engagement in political activities such as attending political protests, participating in election rallies (Yes/No). Question on political participation were not asked to those aged below 15 and they were coded as having not participated in any political activities in the analysis.
Participation in adolescent programmes	Ever attended family life education program, village health and sanitation days/ adolescent health days or school health programme (Yes/No). Question on family life education was asked to those who were aged 13 and above at both waves, the question on school health programme was asked to those who were enrolled in school at the time of the survey at both waves and the question on village health and nutrition day was asked to rural respondents at wave 1 and the question was rephrased to adolescent health days and asked to both rural and urban respondents at wave 2. We also note that the reference period used varied—the question on family life education measured whether the respondent ever attended at wave 1 and in the three years preceding the interview at wave 2, while the questions on school health programme and village health and sanitation days used the reference period of one year preceding the interview at both waves.
Interaction with frontline workers	Interacted with frontline health workers such as Accredited Social Health Activists or Anganwadi workers in the year preceding the interview (Yes/No)
**Societal institutions and influences**	
Education	Years of schooling successfully completed (range 0–17)
Engagement in paid work	Engaged in paid work in the year preceding the interview (Yes/No)
Use of digital media	Used internet or social media (Yes/No). Questions on internet and social media access were asked to those who reported at least 5 years of schooling; those with less than 5 years of schooling were considered not to have accessed internet or social media for the analysis presented in this paper

**Table 2:** Selected background characteristics of study participants at wave-1, 2015–16.

Background characteristics	Boys aged 10–14 at wave 1	Boys aged 15–19 at wave 1	Girls aged 10–14 at wave 1	Girls aged 15–19 at wave 1
**Place of residence**				
Urban	16.3	19.2	18.3	18.3
Rural	83.7	80.8	81.7	81.7
**Religion**				
Hindu	85.1	84.6	78.5	76.9
Muslim/Others	14.9	15.4	21.5	23.1
**Caste**				
Scheduled castes/tribes	25.7	27.9	25.6	22.9
Other backward castes	56.7	54.4	56	54.7
General castes	17.5	17.7	18.4	22.4
**Completed years of schooling**				
None	1.5	2.9	3.9	6.5
1–4 years	36.2	2.8	29.0	3.1
5–9 years	62.3	53.6	67.0	46.6
10+ years	0.0	40.7	0.0	43.8
**N**	**1712**	**2716**	**1439**	**6168**

### Data analysis

We used univariate and bivariate analyses to examine changes in adolescents’ gender role attitudes over time and the association between explanatory variables and gender role attitudes disaggregated by sex and age. We used linear fixed effects regression models to identify factors that shape gender role attitudes among younger and older adolescent girls and boys. We opted for fixed-effects regression because it leverages the longitudinal structure of the data and estimates within-subject differences in the outcome variable–gender role attitudes in our paper–over time as a function of within-subject differences in explanatory variables. It also addresses endogeneity by controlling for time invariant characteristics of the respondents or their environments (e.g., caste, religion, sex), and thereby, eliminates omitted variable bias [35]. The Hausman test results confirmed that fixed effects model was more appropriate than the random effects model for our analysis.

We fitted two fixed effects models–one in which we included age (see Table 4) and one in which we excluded it (see S2 Table).

To investigate associations between GRA and explanatory variables (*X*_*it*_) status, while controlling for other variables (*C*_*it*_), we used the following fixed effects (FE) regression model:

$$GRA_{it}=\beta_{0}+\beta_{1}X_{1t}+\beta_{2}X_{2t}+\ldots ..+\beta_{k}X_{2t}+\beta_{c}C_{it}+ai+uit$$

where i is the respondent, t is the time (wave-1 and wave-2), β0 is constant term, β is fixed effect regression coefficients, a is an unobserved individual effect; and u is a random error. The *X*_1_, *X*_2,_…*X*_k_ are explanatory variables used in the model (please refer Table 1 for the list of variables). *C*_*it*_ represents other variables (e.g. age) that we controlled for.

We fitted separate models for younger and older boys and girls in order to capture differences in predictors of gender role attitudes across these four sub-groups of adolescents. All analyses were conducted using STATA software (version 15.0).

## Results

### Descriptive results

Most study participants were from rural areas (81–84%) and belonged to Hindu religion (77–85%) (Table 2). More than half of the respondents belonged to other backward castes (54–57%) and a quarter belonged to scheduled castes or tribes (23–28%). Almost all had completed at least 5 years of schooling (62–67% of younger adolescents, and 90–94% of older adolescents). The mean household wealth index score ranged from 22.0 to 24.2 out of 57.

Table 3 presents change over time in the explanatory variables and shows that while some variables showed an increase over time, others declined or remained same. Specifically, the proportion of adolescents who thought about the profession or vocation that they would like to take up when they reach adulthood (aspirations) increased over time across all categories of adolescents (by 8–14 percentage points) (p<0.001). Similarly, adolescents who reported five or more friends increased, with boys reporting the highest increase (20–21 percentage points among boys and 7–8 percentage points among girls) (p<0.001). Adolescents who ever used internet or social media increased by 26–48 percentage points over time (p<0.001), with a larger increase among boys than girls (42–48 percentage point increase among boys and 26–31 percentage point increase among girls). While interaction with frontline workers remained unchanged among younger and older boys, it increased by 8 percentage points among younger girls (p = 0.001) and 15 percentage points among older girls (p<0.001). In comparison, parent-child communication decreased over time, with older adolescents reporting the highest decline (older boys: 17 percentage points and older girls: 26 percentage points) (p<0.001). Participation in adolescent programmes was more or less same among younger adolescents while there was 15–23 percentage points decline among older adolescents over time (p<0.001). Reports of experiencing gender discriminatory practices in the household changed very little over time (1–7%) for all sub-groups of adolescents. Reporting of engagement in paid work in the year preceding the interview changed marginally during the inter-survey period, with gender differences persisting among older adolescents (44% of boys versus 25% of girls having engaged in paid work at wave 2). Participation in political activities, likewise, increased marginally over time and gender differences continued (28% of older boys compared to 6% of older girls reporting political participation at wave 2). Finally, the proportion of adolescents reporting having a role model and group membership remained almost unchanged.

**Table 3:** Summary statistics for explanatory variables used in the analysis at wave 1 and wave 2.

	Boys, aged 10–14, unmarried at wave 1			Boys, aged 15–19, unmarried at wave 1			Girls, aged 10–14, unmarried at wave 1			Girls, aged 15–19, unmarried at wave 1		
	Percent or Mean (SD)			Percent or Mean (SD)			Percent or Mean (SD)			Percent or Mean (SD)		
Variables	Wave 1	Wave 2	p-value	Wave 1	Wave 2	p-value	Wave 1	Wave 2	p-value	Wave 1	Wave 2	p-value
Age (Mean)	11.9(1.4)	14.9(1.4)	p<0.001	16.7(1.3)	19.7(1.4)	p<0.001	12.1(1.4)	15.1(1.5)	p<0.001	16.6(1.3)	19.6(1.4)	p<0.001
Education (Mean years)	5.1(2.1)	7.6(2.3)	p<0.001	8.9(2.7)	10.4(3.2)	p<0.001	5.2(2.2)	7.5(2.6)	p<0.001	8.6(3.2)	10.0(4.0)	p<0.001
Household wealth (Mean score)	22.2(8.8)	21.6(8.3)	0.242	24.0(8.6)	24.3(8.5)	0.413	22.0(8.8)	21.7(8.9)	0.902	23.9(9.0)	24.2(9.1)	0.073
Engagement in paid work (%)	17.3	16.1	0.472	46.7	44.4	0.098	13.8	14.5	0.647	24.5	24.8	0.732
Aspirations (%)	71.4	85.3	p<0.001	78.3	90.4	p<0.001	61.5	71.5	p<0.001	67.7	75.7	p<0.001
Parent-child communication (%)	82.6	76.2	p<0.001	70.2	53.0	p<0.001	86.3	84.1	0.207	83.6	57.9	p<0.001
Gender discriminatory experience at home (%))	7.8	9.1	0.353	7.3	6.4	0.344	15.2	8.5	p<0.001	12.0	5.0	p<0.001
Peer network size (%)	27.5	47.5	p<0.001	33.6	54.2	p<0.001	30.2	38.3	p<0.001	28.1	34.6	p<0.001
Role models (%)	37.7	43.2	0.016	46.0	46.3	0.893	36.7	43.0	0.014	35.8	35.0	0.612
Group membership (%)	5.1	4.3	0.547	8.9	4.7	p<0.001	2.2	2.7	0.578	5.0	2.9	p<0.001
Political participation (%)	0.0	13.2	-	22.5	28.2	0.003	0.0	4.2	-	5.2	6.1	0.188
Participation in adolescent programmes (%)	22.5	23.7	0.638	21.0	6.4	p<0.001	26.1	24.6	0.442	27.6	4.5	p<0.001
Interaction with frontline workers (%)	10.4	11.8	0.392	4.9	8.8	0.001	11.7	19.6	0.001	10.1	25.3	p<0.001
Use of digital media (%)	8.8	57.0	p<0.001	38.0	79.9	p<0.001	2.0	28.2	p<0.001	8.7	40.1	p<0.001
N	**1712**	**1712**		**2716**	**2716**		**1439**	**1439**		**6168**	**6168**	

### Changes in gender role attitudes over time (bivariate results)

**Fig 2:**
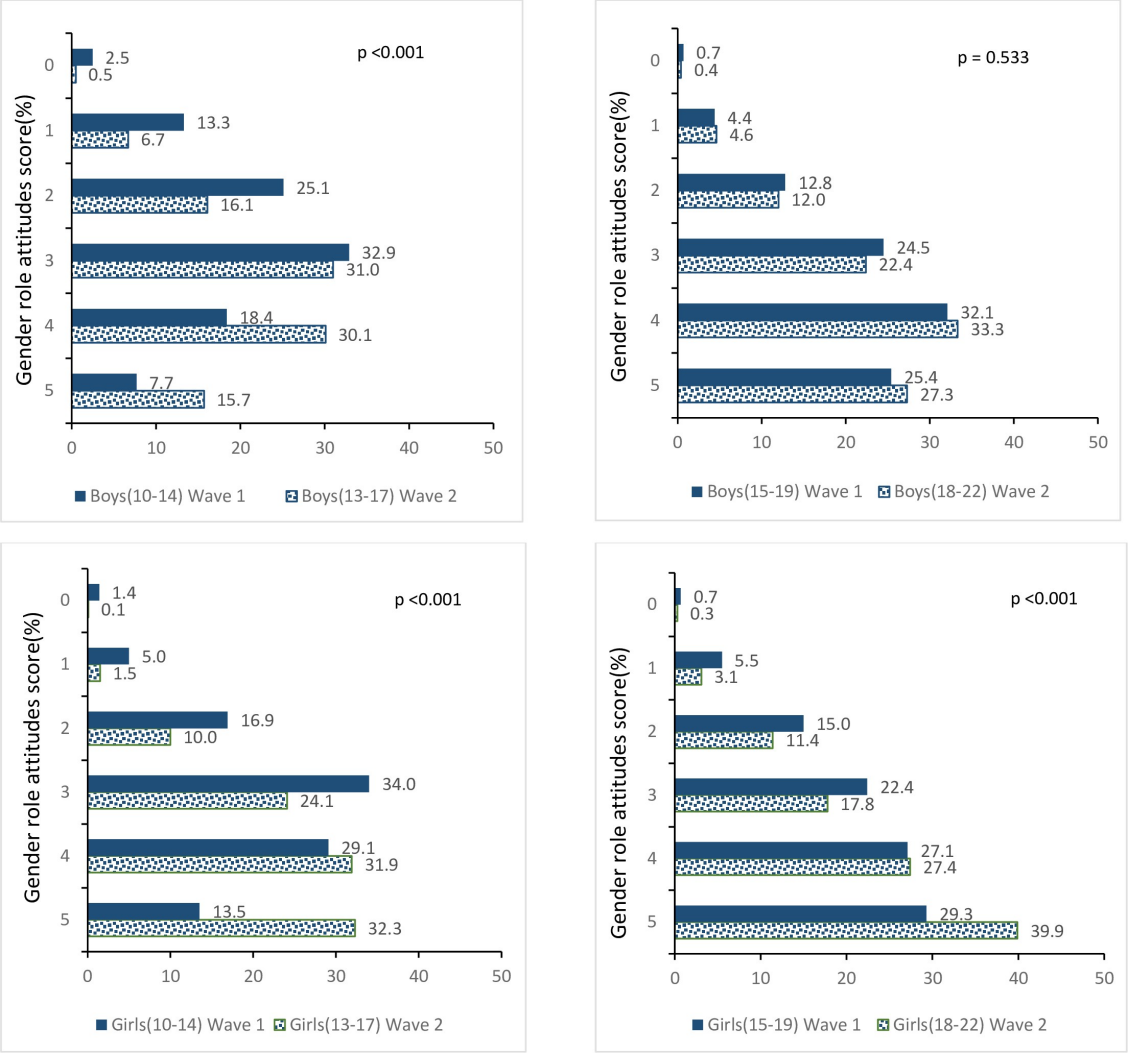
Change in gender role attitudes score among adolescents over time, wave 1 (2015–16) and wave 2 (2018–19).

Younger adolescents, that is, boys and girls who were aged 10–14 at wave 1, became more egalitarian over time (p<0.001; Fig 2). Younger adolescent girls were significantly more likely to hold gender egalitarian attitudes–i.e., scoring 4 or 5 on the gender attitude index–than younger boys at both wave 1, when participants were 10–14, and wave 2, when participants were 13–17 years old (42.6% of girls vs. 26.1% of boys at wave 1, p <0.001; 64.2% of girls vs. 45.8% of boys at wave 2, p <0.001). The proportion of younger adolescents who scored 4 or 5 on the index of gender role attitudes increased by 20–22 percentage points among boys and girls (from 26% at wave 1 to 46% at wave 2 among boys and from 43% at wave 1 to 64% at wave 2 among girls). Gender role attitudes became more egalitarian over time among girls who were aged 15–19 at wave 1 as well–those who scored 4 or 5 on the index of gender role attitudes increased by 11 percentage points by wave 2 when participants were 18–22 years old (from 56% at wave 1 to 67% at wave 2; p<0.001). However, gender role attitudes hardly changed over time among boys who were aged 15–19 at wave 1 –those who scored 4 or 5 on the index of gender role attitudes remained similar in both waves—58% at wave 1 and 61% at wave 2.

### Changes in gender attitudes over time (regression results)

Controlling for other explanatory variables, our findings show that the GRA score significantly improved with age among adolescents with the exception of older boys. With increasing age, the GRA score improved by 0.14 unit among younger boys (p<0.001) and 0.19 unit among younger girls (p<0.001) and 0.08 unit among older girls (p<0.001). In contrast, the GRA score became less egalitarian with increasing age among older boys (p<0.10).

### Factors that shape gender role attitudes

Table 4 presents the results of the fixed effects regression analysis of factors that promote or inhibit egalitarian gender role attitudes among adolescent boys and girls. Younger and older girls who had aspirations for their lives, i.e., articulated the profession or vocation that they would like to pursue in adulthood, were more egalitarian compared to those who did not (**β** = 0.16, p<0.01 for younger girls and **β** = 0.16, p<0.001 for older girls). Articulation of future aspirations was not associated with gender role attitudes among younger or older boys in the full model; however, it was associated with egalitarian attitudes among younger boys in the model in which age was excluded (**β** = 0.14, p<0.05; S2 Table).

**Table 4:** Estimated effects of explanatory variables on the gender role attitudes from linear fixed-effects models.

Indicators	Boys aged 10–14 at wave 1	Boys aged 15–19 at wave 1	Girls aged 10–14 at wave 1	Girls aged 15–19 at wave 1
Age	0.14***	-0.03#	0.19***	0.08***
	(0.08–0.20)	(-0.06–0.01)	(0.14–0.24)	(0.05–0.10)
Education	0.02	0.06**	-0.02	0.03*
	(-0.04–0.08)	(0.02–0.10)	(-0.08–0.03)	(0.003–0.06)
Engagement in paid work	-0.005	0.10*	0.09	0.02
	(-0.15–0.14)	(0.002–0.17)	(-0.07–0.26)	(-0.04–0.09)
Aspirations	0.09	-0.02	0.16**	0.16***
	(-0.04–0.22)	(-0.12–0.09)	(0.04–0.28)	(0.10–0.22)
Parent-adolescent communication	0.08	-0.02	0.41***	0.07*
	(-0.07–0.17)	(-0.11–0.07)	(0.24–0.58)	(0.001–0.13)
Gender discriminatory practices at home	-0.17#	-0.08	-0.11#	-0.03
	(-0.37- -0.03)	(-0.23–0.08)	(-0.29- -0.06)	(-0.06–0.12)
Household wealth	0.004	-0.001	0.003	0.001
	(-0.005–0.01)	(-0.007–0.006)	(-0.01–0.01)	(-0.002–0.01)
Peer network size	0.05	-0.01	0.07	0.02
	(-0.08–0.20)	(0.09–0.07)	(-0.04–0.18)	(-0.04–0.07)
Role models	0.07	0.10*	0.11#	0.03
	(-0.03–0.17)	(0.01–0.17)	(-0.01–0.22)	(-0.03–0.08)
Group membership	-0.26*	-0.11	-0.16	0.01
	(-0.48- -0.03)	(-0.25–0.03)	(-0.47–0.14)	(-0.12–0.13)
Political participation	0.15	-0.02	-0.17	-0.06
	(-0.05–0.35)	(-0.10–0.07)	(-0.50–0.15)	(-0.16–0.04)
Participation in adolescent programs	-0.02	0.05	0.23***	0.12***
	(-0.13–0.10)	(-0.06–0.16)	(0.11–0.34)	(0.06–0.19)
Interaction with frontline workers	0.01	-0.10	0.16*	0.04
	(-0.16–0.18)	(-0.27–0.07)	(0.01–0.31)	(-0.03–0.11)
Use of digital media	-0.05	0.20***	0.13#	0.12***
	(-0.19–0.08)	(0.10–0.30)	(-0.02–0.28)	(0.06–0.19)
F-stat	15.18***	3.36***	24.55***	26.36***
F-test for individual effects	1.43***	1.55***	1.36***	1.50***
Rho	0.45	0.46	0.44	0.47
Hausman test: Chi-square	76.32***	109.77***	55.98***	211.73***
**N**	**1712**	**2716**	**1439**	**6168**

Note: Values in the parentheses show confidence intervals

*** p <0.001

** p<0.01

* p <0.05

# p<0.10.

Younger and older girls who reported having discussed issues relevant to adolescents, such as school performance, friendship, being teased or bullied, physical changes during adolescence, or reproduction with their parents in the year preceding the interview were more egalitarian than their counterparts who had not (**β** = 0.41, p<0.001 for younger girls and **β** = 0.07, p<0.05 for older girls). However, parent-adolescent communication was not associated with gender role attitudes among younger or older boys. Findings also show that younger boys who reported that their parents favoured them over their sisters, as well as younger girls who reported that their parents discriminated against them, were less likely to be gender egalitarian, compared with, those who did not report such discriminatory experiences (**β** = -0.17, p<0.10 for younger boys and **β** = -0.11, p<0.10 for younger girls). We did not find any relationship between household wealth and adolescents’ gender role attitudes.

The size of adolescents’ peer networks did not influence gender role attitudes among any sub-group of adolescents. Older boys and younger girls who reported that they had a role model in their life reported more egalitarian attitudes compared with those who did not have a role model (**β** = 0.10, p<0.05 for older boys and **β** = 0.11, p<0.10 for younger girls), while no such association was observed for younger boys or older girls.

While relatively few participants reported group membership, including self-help groups, sports clubs, and groups formed under government programmes, group membership had a negative effect on younger boys’ gender role attitudes; GRA score was lower by 0.26 units among younger boys (p<0.05) with membership in groups. We found no association between adolescents’ participation in political activities and their gender role attitudes. However, participation in political activities was positively associated with gender egalitarian attitudes among younger boys in the model in which age was excluded (**β** = 0.22, p<0.05; S2 Table)

Participation in adolescent programs such as life skills education, school health programmes and village health and nutrition days/ adolescent health days had a significant positive influence on girls’ gender role attitudes (**β** = 0.23, p<0.001 for younger girls and **β** = 0.12, p<0.001 for older girls). We did not find any such association for younger or older boys. Interaction with frontline workers was highly associated with egalitarian gender role attitudes among younger girls in the full model (**β** = 0.16, p<0.05). It was also associated with the gender role attitudes of younger girls as well as older girls in the reduced model in which we excluded age (younger girls: **β** = 0.20, p<0.01 and older girls: **β** = 0.09, p<0.05).

Findings show that years of schooling completed had a significant positive effect on egalitarian gender role attitudes of older boys and girls, although the regression coefficients were small (**β** = 0.06, p<0.01 for older boys and **β** = 0.03, p<0.05 for older girls), while we found no such relationship for younger boys and girls. We note, however, in the model in which we excluded age, years of schooling had a positive effect on the gender role attitudes of all adolescents, regardless of their age and sex–i.e., with an additional year of schooling, GRA score was higher by 0.14 units among younger boys, 0.15 units among younger girls, 0.03 units among older boys and 0.10 units among older girls (S2 Table). Engagement in paid economic activities was positively associated with egalitarian gender role attitudes among older boys (**β** = 0.10, p<0.05), but no such relationship was observed for younger boys and girls or for older girls.

Finally, adolescents (except younger boys) who used internet or social media were more likely to express gender egalitarian attitudes than those who had not. This relationship was statistically significant for older boys (**β** = 0.20, p<0.001), younger girls (**β** = 0.13, p<0.10) and older girls (**β** = 0.12, p<0.001) in the full model. The same pattern is also seen in the reduced model, but with larger regression coefficients for girls (older boys: **β** = 0.17, p<0.001; younger girls: **β** = 0.20, p<0.01; older girls: **β** = 0.20, p<0.001).

## Discussion

Our study provides rigorous evidence on the mutability of gender role attitudes among a representative longitudinal cohort of boys and girls in two large states of India, and shows that multiple factors determine the gender role attitudes of adolescents and of young adults. Gender role attitudes became more egalitarian over time among boys and girls, except among the older cohort of boys in our study. Findings also show that among both younger and older cohorts, girls/young women held more egalitarian views than boys/young men and that this pattern held over time for both cohorts. Factors that influenced gender role attitudes differed for younger and older adolescents, particularly among boys. While some predictors differed for boys and girls, there were substantial similarities as well. Gender attitudes were affected by factors at the individual, family, peer, and societal levels, as well as by community engagement.

Social influences included education, work, and digital media access. Several studies have observed the effect of education on gender role attitudes [36, 37], although some have noted that increased schooling does not always enhance gender egalitarian views [38, 39]. In our study, educational attainment led to significantly more equitable gender attitudes among older girls and older boys, however, effect sizes were relatively small. The lack of an effect for younger adolescents may be due to school exerting a conforming role at younger ages, or it may be due to effects only accruing once secondary school is reached. Although it is argued that men’s work status is unlikely to have any effect on their gender attitudes given that male employment is the norm [36], our analysis shows that older boys who had engaged in paid work were more likely than others to uphold gender egalitarian attitudes. Work had no effect on girls’ or younger boys’ gender attitudes. Among the factors with the greatest impact was digital media access, which led to significantly more equitable norms among younger and older girls and older boys. A systematic review of factors underlying gender role attitudes in early adolescence noted that the evidence on the relationship between media and gender role attitudes was inconclusive and called for additional research to better understand this relationship [17]. While on the one hand, online media may serve as a platform to reinforce offline stereotypical gender norms, on the other, they may challenge existing gender norms and expose young people to alternate gender identities and norms [40, 41]. Our findings conform to the latter view and show that adolescents, with the exception of young adolescent boys, who accessed the internet and social media were more likely than those who did not to uphold gender egalitarian views.

Youth participation and engagement is another factor that affected gender attitudes. However only participation in adolescent programs and interaction with frontline health workers were significant in the expected direction, and then only so for girls. Group membership, which few adolescents reported, affected only younger boys’ gender attitudes and shifted them to be less egalitarian. In India, several programmes have been implemented by government and non-governmental organizations to raise adolescents’ awareness about reproductive and sexual health matters, facilitate their access to reproductive and sexual health counselling and services, address gender disparities, and build a supportive environment. However, the reach of these programmes remains uneven and their impact has not been rigorously tested [42]. Our study included a limited set of questions to explore adolescents’ participation in some of these programmes, for example, attendance in life skills education programmes, school health programme and adolescent health days, and young people’s interactions with frontline health workers. Our findings show that participation in adolescent programs and interactions with frontline health workers were significantly associated with egalitarian attitudes among girls. Although not exactly comparable, reviews of life skills/sexuality education/safe spaces programmes have shown that these programmes are useful for modifying adolescents’ gender role attitudes [43, 44]. For both indicators, effect sizes were higher for younger girls than for older girls, and only participation in adolescent programs was significant for both. Findings that neither participation in adolescent programmes nor interactions with frontline health workers were associated with boys’ gender role attitudes may be because adolescent programmes have, justifiably, been directed towards empowering and broadening the horizons of girls, and less on promoting positive notions of masculinity.

Peer-level effects were also mixed. Peer network size had no effect on gender attitudes for adolescent girls or boys. Having a role model, however, had a significant impact among some adolescents. Exposure to and interactions with role models who demonstrate counter-stereotypical notions and practices may encourage adolescents to accept a broader range of roles for women and men [17, 45]. Our findings show that having a role model (such as family members/relatives, teachers, professionals, friends, army/police, sports personalities, friends, actors, politicians and others) enhanced the egalitarian attitudes of older boys and younger girls.

Family-level influences also had mixed effects. We found no effects for household wealth. The intergenerational transmission of gender ideologies is well documented in the global literature [23, 37, 46]. Such transmission may take place through direct interaction, observation and modeling and the construction of home environment [47]. Our study also highlights the role of parental factors on gender role attitudes of adolescents. We found that younger boys and girls who reported gendered discriminatory practices at home (favouring sons and discriminating against daughters) were less likely than others to report gender egalitarian views. The fact that no such relationship was observed for older adolescents supports the argument that the family-of-origin effect is likely to be weakened as peer influences and own life experiences become stronger during the transition to adulthood [48]. We also found that younger and older girls (but not boys) who reported discussing topics like school performance, friendship, bullying/teasing, and physical changes were more likely than their counterparts who did not report such parent-adolescent communication to express egalitarian gender role attitudes. We speculate that in settings like India where parent-adolescent communication is limited [18, 33, 34, 42, 49, 50], parents who discussed these topics with their adolescent daughters may be gender egalitarian in their attitudes, and thus, may have influenced their daughter’s gender role attitudes. Studies have also shown that boys and girls tend to talk more to their mothers than fathers [18, 33, 34] and mothers’ gender role attitudes were more likely to be associated with daughters’ attitudes than with sons’ [51, 52]; this perhaps may be the reason for the absence of an association between parent-child communication and boys’ gender role attitudes in our study. However, our study did not collect data on parental gender role attitudes, and future research is needed to better understand the pathways through which parent-child communication affects the gender role attitudes of girls.

Individual level factors assessed included age and aspirations. While the relationship between adherence to stereotypical gender roles and adolescents’ educational and occupational aspirations is established in several studies [45, 53, 54], the reverse relationship is not reported in any of these studies. Our findings show that having career aspirations for adulthood was significantly associated with gender egalitarian views among younger and older girls, but not among boys. As argued by Nathan (2005), “aspirations can result in the creation of new needs, introduce new ways of living into a person’s preference set or change the relative valuation of different ways of living” [55]. Girls in our study who expressed future aspirations may have had “more interactions within their own peer networks and outside, exposure to other practices and ways of living, access to and control over resources and knowledge that can help develop the capacity to aspire,” leading to the creation of new needs and the development of new capabilities, including critically challenging beliefs and norms that can constrain their realization of their aspirations. Alternatively, girls’ expanded aspirations for their careers may signal an early expression of more egalitarian beliefs in what is possible for girls–which over time translates to more egalitarian gender attitudes. For boys, aspiring to a job is consistent with prevailing gender norms, which may explain why career aspirations had no effect on boys’ gender attitudes.

Finally, gender attitudes improved significantly with age for younger boys and younger and older girls although effect sizes were substantially higher among younger than older adolescent girls. Moreover, among boys, for older boys, age predicted less equitable attitudes, though the effect size was small. Why age has an independent effect on gender attitudes may be due to adolescents’ cognitive development [56]. For example, as their capacity for reasoning and critical thinking expand, they may be better able to hold independent views on gender roles and norms. Alternatively, it could be that with age there is less pressure (or greater resistance or immunity to pressure) to conform, and thus greater openness to shifting gender attitudes. These reasons might also align with the smaller effect sizes at older ages in that such cognitive developmental stages–and also their benefits–eventually plateau. For older boys, it may be that because they benefit from existing gender norms that they are less inclined to change their attitudes. Our findings suggest that there are different patterns of gender attitude formation for girls and for boys.

Looking across levels and respondents, several important observations are worth noting. Not only are girls more likely to hold gender equitable attitudes than boys of the same age, but a greater number of factors influence their gender attitudes. Younger girls are particularly amenable to change, with eight factors significant in the expected direction, compared to only two for younger boys. Further, we note that factors from all levels of ecosystem influence adolescents’ gender attitudes. For girls, aspirations, age, communication with parents, participation in adolescent programs, and digital media access are significant for both younger and older girls, and particularly strong. For boys, there is less consistency, with digital media the strongest factor for older boys, and for younger boys, age and group membership (though the latter not in the anticipated direction). Participation in adolescent programs does not foster more equitable views among boys, indicating a gap in their content/ relevance for transforming masculinity norms.

While the study findings offer several important insights into gender role attitudes and how they change, these findings must be cautiously interpreted given the study’s limitations. First, gender role attitudes, and other indicators are self-reported, which means that they are susceptible to social desirability and recall biases. Second, the study findings are only representative of adolescents in Bihar and UP–hence these findings may not be generalizable to adolescents in other places across the globe, or even across India. Third, we acknowledge that the Cronbach’s alpha was not very strong; it may be partially due to the small number of questions available for constructing the index. Nonetheless, as a composite score of gender attitude questions that cover different types of gender norms, it does provide a measure of respondents’ beliefs. Fourth, the index used in this analysis does not include gender attitudes related to intimate partner violence. It could be that inclusion of such questions would lead to different results. Finally, as noted in the study design section, there was some attrition bias between survey rounds.

This longitudinal study finds that the gender attitudes of adolescent girls and boys in Bihar and UP become more equitable over time, and that girls, regardless of age, hold more equitable gender views than boys of the same age. While improvements in gender attitude scores are greater for younger adolescents, they also improve among older adolescent girls, with only older boys showing relative stability from wave 1 to wave 2. We also identify multiple factors that shape adolescents’ gender attitudes, with distinct variation by both sex and age. These factors are important to consider when developing programs that aim to improve gender equity.

## Supporting information

S1 Table The characteristics at wave 1 of adolescents who were re-interviewed and who were not. (DOCX)

S2 Table Estimated effects of explanatory variables, excluding age, on the gender role attitudes from linear fixed effects models. (DOCX)
