# Supplementary material for: What shapes gender attitudes among adolescent girls and boys? Evidence from the UDAYA Longitudinal Study in India
Source: PLoS One. 2021 Mar 18;16(3):e0248766. doi: 10.1371/journal.pone.0248766 (PMC7971892; doi:10.1371/journal.pone.0248766)
Supplement: S2 Table — (DOCX) [file pone.0248766.s002.docx]

**S2 Table. Estimated effects of explanatory variables, excluding age, on the gender role attitudes from linear fixed effects models**

| **Indicators** | **Boys aged 10-14 at wave 1** | **Boys aged 15-18 at wave 1** | **Girls aged 10-14 at wave 1** | **Girls aged 15-18 at wave 1** |
| --- | --- | --- | --- | --- |
| Wealth score | 0.004(-0.005-0.014) | -0.0004(-0.007-0.006) | 0.004(-0.006-0.01) | 0.002(-0.002-0.006) |
| Education | 0.14***  (0.10-0.17) | 0.03*  (0.003-0.06) | 0.15***  (0.11-0.18) | 0.10***  (0.07-0.12) |
| Engagement in paid work | 0.01  (-0.14-0.16) | 0.09*  (0.005-0.17) | 0.07  (-0.10-0.24) | 0.03  (-0.04-0.10) |
| Aspirations | 0.14*  (0.008-0.26) | -0.04  (-0.14-0.08) | 0.22***  (0.10-0.35) | 0.19***  (0.13-0.25) |
| Parent-adolescent communication | 0.04  (-0.11-0.18) | -0.01  (-0.10-0.08) | 0.36***  (0.19-0.54) | -0.01  (-0.07-0.05) |
| Gender discriminatory practices at home | -0.17#  (-0.36- -0.03) | -0.07  (-0.23-0.08) | -0.15#  (-0.32-0.02) | -0.003  (-0.09-0.08) |
|  |  |  |  |  |
| Peer network size | 0.09  (-0.03-0.20) | -0.02  (-0.10-0.06) | 0.09  (-0.02-0.21) | 0.03  (-0.03-0.08) |
| Role models | 0.07  (-0.04-0.17) | 0.09*  (0.01-0.17) | 0.11#  (-0.01-0.22) | 0.03  (-0.03-0.08) |
| Group membership | -0.28*  (-0.51- -0.05) | -0.11  (-0.25-0.04) | -0.14  (-0.45-0.24) | -0.02  (-0.14-0.11) |
| Political participation | 0.22*  (0.02-0.42) | -0.02  (-0.10-0.07) | -0.09  (-0.42-0.24) | -0.07  (-0.16-0.04) |
| Participation in adolescent programs | -0.03  (-0.15-0.08) | 0.06  (-0.05-0.16) | 0.21***  (0.10-0.33) | 0.08*  (0.01-0.14) |
| Interaction with frontline workers | 0.01  (-0.17-0.19) | -0.10  (-0.27-0.06) | 0.20**  (0.05-0.35) | 0.09*  (0.02-0.15) |
| Use of digital media | 0.02  (-0.11-0.15) | 0.17***  (0.08-0.26) | 0.20**  (0.04-0.34) | 0.20***  (0.12-0.25) |
| F-stat | 14.41*** | 3.42*** | 21.65*** | 23.70*** |
| F-test for individual effects | 1.42*** | 1.55*** | 1.30*** | 1.47*** |
| Rho | 0.44 | 0.47 | 0.41 | 0.44 |
| Hausman test: Chi-square | 60.39*** | 112.41*** | 40.95*** | 167.69*** |
|  |  |  |  |  |
| **N** | **1712** | **2716** | **1439** | **6168** |

Note: Values in the parentheses show confidence intervals; *** p <0.001, ** p<0.01, * p <0.05, # p<0.10.
